# Aphthous Sore Mouth of Infants

**Published:** 1882-02

**Authors:** 


### APHTHOUS SORE MOUTH OF INFANTS.

Prof. Wallace believes that the sodium sulphite solution is the best remedy for aphthous sore mouth for infants.

℞. Sodii sulphit , gr. xxx.

Glycerini,

Aquæ, aa ℥ss. M.

To be used on a swab every two hours. Where the child is using a nursing bottle, scrupulous cleanliness is required. The rubber nipple should be turned inside out after each time of using, washed clean, and placed in a solution of bicarbonate of sodium (baking soda), in a tumbler, until again needed. It is better to have two, and use them alternately. Milk must never be allowed to stand in the nursing bottle until it becomes sour.---*College and Clinical Record.*

Mental Pathology: "Don't you think," asked Brown, "that Pingrey is laboring under a hallucination?" "No, I don't," replied Fogg; "he has a hallucination, but I don't believe he is laboring under it. I never knew him to labor under anything.---*Boston Transcript.*

